# Glycerophospholipid and Sphingolipid Species and Mortality: The Ludwigshafen Risk and Cardiovascular Health (LURIC) Study

**DOI:** 10.1371/journal.pone.0085724

**Published:** 2014-01-17

**Authors:** Alexander Sigruener, Marcus E. Kleber, Susanne Heimerl, Gerhard Liebisch, Gerd Schmitz, Winfried Maerz

**Affiliations:** 1 Institute for Laboratory Medicine and Transfusion Medicine, Regensburg University Medical Center, Regensburg, Germany; 2 Medical Clinic V, Mannheim Medical Faculty, University of Heidelberg, Mannheim, Germany; 3 Clinical Institute of Medical and Chemical Laboratory Diagnostics, Medical University of Graz, Graz, Austria; 4 Synlab Academy, Synlab Services GmbH, Mannheim, Germany; University of Milano, Italy

## Abstract

Vascular and metabolic diseases cause half of total mortality in Europe. New prognostic markers would provide a valuable tool to improve outcome. First evidence supports the usefulness of plasma lipid species as easily accessible markers for certain diseases. Here we analyzed association of plasma lipid species with mortality in the Ludwigshafen Risk and Cardiovascular Health (LURIC) study. Plasma lipid species were quantified by electrospray ionization tandem mass spectrometry and Cox proportional hazards regression was applied to assess their association with total and cardiovascular mortality. Overall no differences were detected between total and cardiovascular mortality. Highly polyunsaturated phosphatidylcholine species together with lysophosphatidylcholine species and long chain saturated sphingomyelin and ceramide species seem to be associated with a protective effect. The predominantly circulating phosphatidylcholine-based as well as phosphatidylethanolamine-based ether species and phosphatidylethanolamine species were positively associated with total and cardiovascular mortality. Saturated and monounsaturated phosphatidylcholine species, especially phosphatidylcholine 32∶0 (most probably dipalmitoyl-phosphatidylcholine) and palmitate containing sphingomyelin and ceramide species showed together with 24∶1 containing sphingomyelin and ceramide species strongest positive association with mortality. A quotient of the sums of the six most protective species and the six species with the strongest positive mortality association indicated an almost 3-fold increased risk of mortality, which was higher than the hazard ratio for known risk factors in our cohort. Plasma lipid species levels and especially ratios of certain species may be valuable prognostic marker for cardiovascular and total mortality.

## Introduction

Vascular and metabolic diseases represent a major challenge in healthcare in Europe, causing almost half of total mortality [1]. Major non-changeable risk factors are age, gender and heredity. They stand in contrast to modifiable risk factors like hypertension, metabolic overload, physical inactivity, obesity, low high density lipoprotein (HDL) cholesterol and/or high triglycerides (TG) which cluster in the metabolic syndrome or type 2 diabetes mellitus. It is suggested that obesity related metabolic diseases like type 2 diabetes mellitus and its major endpoint cardiovascular disease are the result of a common molecular mechanism marked by two independent mechanisms: 1) selective insulin resistance (IR), and 2) lipotoxicity [2].

Identification of plasma lipid species associated with diseases provided first evidence for their usefulness as easily accessible markers, improving diagnosis and treatment. Recently more than 500 lipids species constituting the main lipid classes have been quantified in human plasma. These data may serve as base for additional studies to analyze their role in health and disease [3]. Initial data indicate that lipid profiling may be helpful to reveal certain diseases like e.g. type 1 diabetes [4], non-alcoholic fatty liver disease [5] and that plasma sphingomyelin (SM) and ceramide (Cer) may be used as potential biomarkers for Alzheimeŕs disease [6]. Furthermore they may be used not only to identify clinical endpoints but also intermediate stages [7].

Zwitterionic phosphatidylcholine (PC) and phosphatidylethanolamine (PE) are the two main phospholipids in eukaryotic cells. PC is also a major component of several secretory products like HDL, bile, and pulmonary surfactant [8]. Enzymatic hydrolysis of one fatty acid (FA) mainly by phospholipase A_2_ activity leads to Lyso-PC (LPC) species formation, acting as highly abundant signaling molecules through specific G protein-coupled receptors [9]. A recent study revealed associations of phospholipase A_2_, LPC, lysophosphatidic acid and proinflammatory cytokine levels in atherosclerotic plaques, suggesting involvement of LPC in plaque inflammation and vulnerability [10].

It was shown recently that total PC and TG are elevated in hypertensive patients and that antihypertensive agents reduce plasma total cholesteryl esters and TG [11]. Obesity leads to increased lipid load in plasma, while IR controlled for body mass index (BMI) has little effects on plasma lipid composition. In hypertension ether lipids, especially arachidonic (20∶4) and docosapentaenoic (22∶5) acid containing PCs and PEs, were found reduced [12]. Arachidonic and docosapentaenoic acid are essential FAs generated from linoleic and α-linolenic acid by desaturation mediated by the fatty acid desaturases 2 (FADS2) and 1 (FADS1) and elongation through ELOVL fatty acid elongase 5 (ELOVL5). A common finding is the association of members of the FADS gene cluster (FADS1-3) with phospholipid and FA species and their chain length and degree of saturation [13]–[15]. FADS1 catalyses delta-5 desaturation (D5D), converting eicosatrienoyl-CoA (20∶3) to arachidonyl-CoA (20∶4). These FAs are found for example in PC 36∶3 and PC 36∶4 and the ratio between PC 36∶3 and 36∶4 is regarded as a good predictor of the FADS1/D5D activity [13]. Furthermore several additional loci associated with phospholipid species were identified, among those key enzymes involved in lipid metabolic processes like beta oxidation, polyunsaturated FA (PUFA) synthesis, TG breakdown and receptor-mediated lipoprotein uptake [13], [15]. An overview of published SNP-lipid species correlations related to vascular and metabolic disease was recently published [16]. The FADS cluster is also associated to SM species formation, where FA chain length and desaturation determine substrate specificity for Cer-synthases regulating the FA composition of Cer [17] from which SM is generated. Sphingolipids are localized in cell and organelle membranes and lipoproteins and are suggested to exert important functions in cell signaling, cell surface protection, protein and lipid transport and sorting. Recent publications highlight their roles in both health and disease including cardiovascular disease [18], [19]. In the EUROSPAN study the FADS cluster and FA specific genes involved in Cer metabolism, namely ceramide synthase 4 (CERS4/LASS4), sphingosine-1-phosphate phosphatase 1 (SGPP1), and serine palmitoyltransferase, long chain base subunit 3 (SPTLC3), were identified as polymorphic genes that correlate with sphingolipid species in plasma [14]. Cer occupies the central position in sphingolipid metabolism. It is either derived from the breakdown of complex sphingolipids via the “salvage pathway” or from “de novo synthesis” at the cytosolic surface of the endoplasmatic reticulum. De novo Cer synthesis shows pronounced specificity for the saturated long chain FAs palmitate (16∶0) and stearate (18∶0) [20]. Both, palmitate and stearate, have been shown to induce apoptosis in cultured cells [21]–[23] and part of cardiomyocyte loss in genesis or progression of heart failure in humans is mediated by apoptosis [24]. As FA-induced apoptosis is specific for these two FAs [22] it has been suggested that FA-induced apoptosis is mediated by Cer synthesis. While this appears to be true for some cell types [22], [23] other data indicate that Cer is not causal for FA-induced apoptosis [25] but may enhance the effect [25]. Cer, IR and inflammation are suggested to be linked via an IL-6 related mechanism. Cer may also influence induction of inflammation that is involved in IR states that are often found with coronary heart disease [26]. Furthermore elevated plasma Cer levels were found to be correlating with hypertension grade [27]. High human plasma SM levels showed association with coronary artery disease (CAD) [28].

Previously, free FA levels were identified as independent predictors of total and cardiovascular mortality and as independent risk factors for future sudden cardiac death in the Ludwigshafen Risk and Cardiovascular Health (LURIC) study [29], [30]. Here we analyzed the association of various plasma lipid species with mortality in the LURIC study. We focused on the analysis of glycerophospholipids, namely PC, LPC, PC O, PE and PE O and two sphingolipids, SM and Cer.

## Materials and Methods

### Study design and participants

The LURIC study design has been described [31]. In brief it included patients of Caucasian origin hospitalized for coronary angiography between June 1997 and May 2001. The study was approved by the ethics review committee at the “Landesärztekammer Rheinland-Pfalz” (Mainz, Germany). Written informed consent was obtained from each of the participants. Clinical indications for angiography were chest pain or non-invasive tests consistent with myocardial ischemia. To limit clinical heterogeneity, individuals suffering from acute illness other than acute coronary syndromes, chronic non-cardiac diseases and a history of malignancy within the five past years were excluded. Criteria for the definition of CAD were recently published [32]. Anthropometric and clinical parameters of the examined cohort were previously published [33] and a short summary is shown in table 1.

**Table 1:** Anthropometric and clinical parameters of the examined cohort.

Age (years)	62.7 (±10.6)
BMI (kg/m2)	27.5 (±4.1)
LDL cholesterol (mg/dL)	116.6 (±34.3)
HDL cholesterol (mg/dL)	38.7 (±10.8)
Triglycerides (mg/dL)	147 (109 – 201)
Mean systolic blood pressure (mmHg)	141.1 (±23.6)
Mean diastolic blood pressure (mmHg)	81.0 (±11.5)
Mean fasting glucose (mg/dL)	113.4 (±35.4)
Male gender	69.7%
CAD (>10% or clinical) (yes/no)	77.9%
CAD 50% or more visual stenosis (yes/no)	67.3%
Diabetics (yes/no)	32.1%
Hypertension (yes/no)	72.7%
Smoking (no/ex(>30d)/active)	35.5%/41.0%/23.4%

BMI, body mass index; CAD, coronary artery disease; HDL, high density lipoprotein; LDL, low density lipoprotein.

### Lipidomics

Plasma lipid species were quantified by electrospray ionization tandem mass spectrometry (ESI-MS/MS) as previously published [34]–[36]. A precursor ion scan of *m/z* 184 specific for phosphocholine containing lipids was used for PC, LPC and SM [34], [36]. Cer was analyzed using a fragment ion of *m/z* 264 [35]. For each lipid class two non-naturally occurring internal standards were added and quantification was achieved by calibration lines generated by addition of naturally occurring lipid species to plasma. Deisotoping and data analysis for all lipid classes was performed by self programmed Excel Macros according to the principles described previously [34]. Lipid species were annotated according to the “Shorthand Notation for Lipid Structures Derived from Mass Spectrometry” [37]. Glycerophospholipid species were annotated based on assumption of even numbered carbon chains only. SM species annotation is based on the assumption that d18∶1 (dihydroxy 18∶1 sphingosine) is the main base of plasma SM species.

### Statistical analysis

All lipid variables were logarithmically transformed and divided by their respective standard deviation to achieve a normal distribution prior to analyses. Cox proportional hazards regression was applied to assess the effect of molecular lipids on total and cardiovascular mortality during a median follow-up of 8 years. The six molecular lipid species which showed the strongest protective effect and the six molecular lipid species that showed the highest risk increment were summed up to create a protective lipids score and a risk lipids score, respectively. We then calculated the ratio of risk lipids to protective lipids and assessed the association with mortality per 0.1 unit increase of the ratio by Cox regression analysis. Models were adjusted for age, gender, smoking, low density lipoprotein (LDL), HDL, diabetes and hypertension. Two-sided P-values <0.05 were used to indicate statistical significance. The SPSS statistical package version 16.0 for Windows was used for all analyses.

## Results

We applied ESI MS/MS to analyze the lipid species of PC, LPC, PE, SM and Cer in plasma of 3316 subjects from the LURIC study.

38 PC, 15 LPC, 30 PC O, 31 PE, 24 PE O, 33 SM and 7 Cer species were detected and associated with total (n = 768) and cardiovascular (n = 484) mortality. Models were adjusted for age, gender, smoking, LDL, HDL, diabetes and hypertension. Our cohort included 733 controls and 2583 CAD positive patients. From the 2583 patients 1368 had survived a myocardial infarction prior to enrollment in the study. The CAD group was significantly older than the control group, showing significantly higher prevalence for type 2 diabetes, smoking, and hypertension. They also had significantly higher TG levels, while HDL and LDL (due to more patients with CAD taking statins) levels were found reduced.

**Figure 1:**
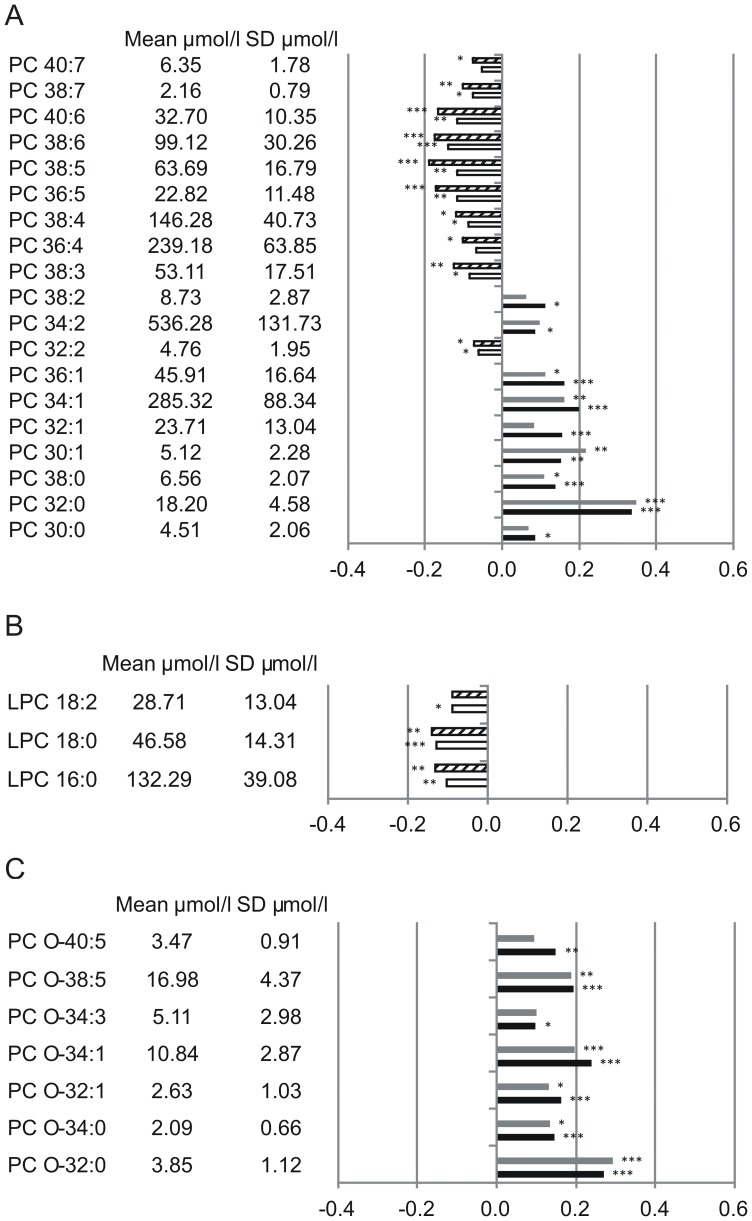
Mortality association of PC species (A), LPC species (B) and PC O species (C). EDTA plasma concentrations were determined by ESI-MS/MS. Species with significant association to CAD and total mortality are shown. Models were adjusted for age, gender, smoking, LDL, HDL, diabetes and hypertension. Bars represent the hazard ratio −1. Positive association with CAD is shown in grey and positive association with total mortality in black. Negative association with CAD is shown by a dashed white bar and negative association with total mortality in white. Species are named according to the number of carbon atoms and degree of desaturation. Besides their association the mean concentration and standard deviation are shown. PC and LPC species were annotated based on assumption of even numbered carbon chains only.

**Figure 2:**
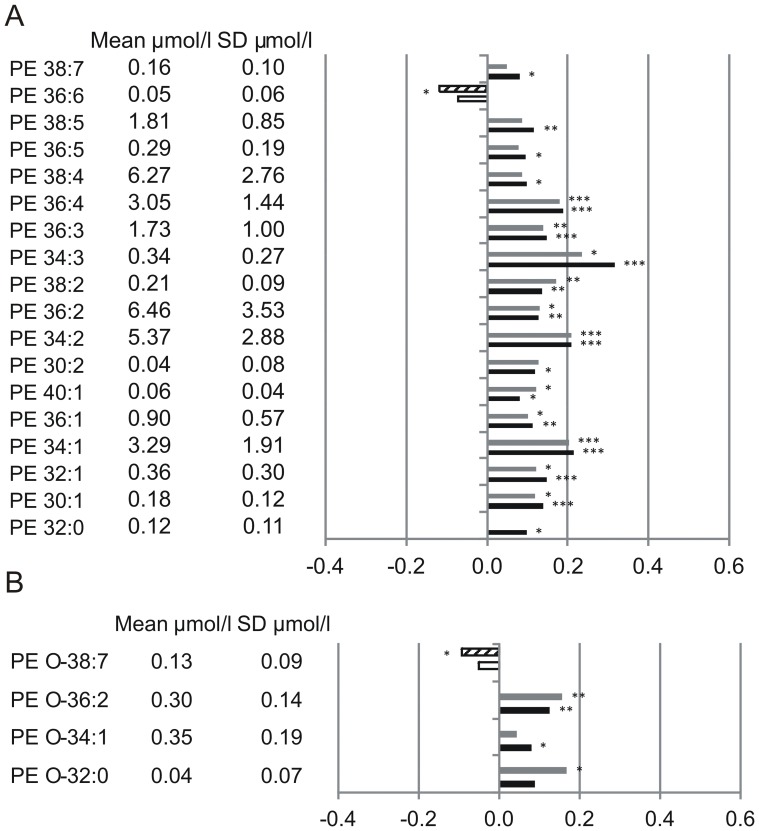
Mortality association of PE species (A) and PE O species (B). Species with significant association to CAD and total mortality are shown. EDTA plasma concentrations were determined by ESI-MS/MS. Models were adjusted for age, gender, smoking, LDL, HDL, diabetes and hypertension. Bars represent the hazard ratio −1. Positive association with CAD is shown in grey and positive association with total mortality in black. Negative association with CAD is shown by a dashed white bar and negative association with total mortality in white. Species are named according to the number of carbon atoms and degree of desaturation. Besides their association the mean concentration and standard deviation are shown. PE species were annotated based on assumption of even numbered carbon chains only.

**Figure 3:**
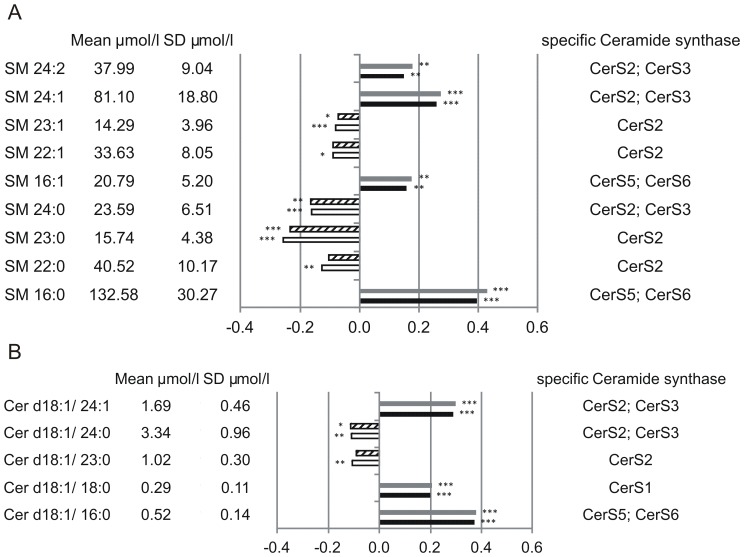
Mortality association of SM species (A) and Cer species (B). EDTA plasma concentrations were determined by ESI-MS/MS. Species with significant association to CAD and total mortality are shown. Models were adjusted for age, gender, smoking, LDL, HDL, diabetes and hypertension. Bars represent the hazard ratio −1. Positive association with CAD is shown in grey and positive association with total mortality in black. Negative association with CAD is shown by a dashed white bar and negative association with total mortality in white. Species are named according to the number of carbon atoms and degree of desaturation. Besides their association the mean concentration and standard deviation are shown together with the putative involved Cer-synthase. SM species annotation is based on the assumption that d18∶1 (dihydroxy 18∶1 sphingosine) is the main base of plasma SM species.

Altogether 19 PC species (Figure 1A), 3 LPC species (Figure 1B), 7 PC O (Figure 1C), 17 PE species (Figure 2A), 5 PE O (Figure 2B), 9 SM species (Figure 3A) and 5 Cer species (Figure 3B) were found significantly associated with total and/or CAD mortality. All identified species were similarly associated with total and CAD mortality (Figure 1–3). Among the identified 19 PC species 9 were positively associated with mortality; three saturated species (PC 30∶0, 32∶0, 38∶0), four monounsaturated species (PC 30∶1, 32∶1, 34∶1, 36∶1) and two polyunsaturated species (PC 34∶2, 38∶2) (Figure 1A). PC 32∶0 showed the strongest positive mortality association. Interestingly PC 32∶1 and PC 32∶2 diminish their mortality association with PC 32∶2 being even slightly, but significantly protective (Figure 1A). In contrast to PC 32∶2, PC 34∶2 and PC 38∶2 did not display any protective effect, although a trend towards lower mortality association compared to their saturated/monounsaturated counterparts is visible. Against this trend PC 30∶1 revealed even stronger mortality association than PC 30∶0. Among the PC 38 species, six desaturation products ranging from PC 38∶2 - PC 38∶7 were identified. While PC 38∶0 and PC 38∶2 were found significantly associated with mortality PC 38∶3 - PC 38∶7 were all significantly associated with a protective effect (Figure 1A). A significant association with a protective effect was also visible for PC 36∶4, PC 36∶5, PC 40∶6 and PC 40∶7. All protective PC species showed overall a slightly higher assocition with death due to CAD than to total mortality. Three LPC species were identified (16∶0, 18∶0, 18∶2) which were all found associated with a protective effect (Figure 1B). All 7 identified PC O species revealed positive mortality association (Figure 1C); two saturated species (PC O-32∶0, PC O-34∶0), two monounsaturated species (PC O-32∶1, PC O-34∶1) and three unsaturated species (PC O-34∶3, PC O-38∶5, PC O-40∶5). Similar to PC 32∶0, PC O-32∶0 showed strongest positive mortality association while mortality association of PC O-32∶1 was reduced.

We identified 17 PE species that associate with mortality with PE 36∶6 being the only protective PE species (Figure 2A). The remaining 16 PE species were positively associated with mortality, independent of chain length and degree of saturation. They consist of one saturated (PE 32∶0), five monounsaturated (PE 30∶1, 32∶1, 34∶1, 36∶1, 40∶1) and 11 polyunsaturated (PE 30∶2, 34∶2, 36∶2, 38∶2, 34∶3, 36∶3, 36∶4, 38∶4, 36∶5, 38∶5, 38∶7) species. PE species of the 34 C-atom series (34∶1, 34∶2, 34∶3) revealed strongest positive mortality association.

From the PE-ether lipid species PE O-32∶0, PE O-34∶1, and PE O-36∶2 showed positive mortality association (Figure 2B). For PE O-38∶7 an assocition with a protective effect was visible (Figure 2B).

Five SM species showed an association with a protective effect, mainly saturated species with 22, 23 and 24 carbon-atoms; a slight association with a protective effect was also visible for SM 22∶1 and 23∶1. In contrast, SM 16∶0, 16∶1, 24∶1 and 24∶2 displayed positive association with mortality (Figure 3A). From the 5 identified Cer species three displayed positive association with mortality (16∶0, 18∶0, 24∶1); the remaining two Cer species (23∶0, 24∶0) were slightly protective (Figure 3B). In summary highly polyunsaturated PC species together with LPC species and long chain saturated SM species seem to be associated with a protective role. PC 32∶0 (probably PC 16∶0/16∶0) and SM 16∶0 showed together with Cer 24∶1 strongest positive association with mortality. Interestingly similar tendencies were observed for 16∶0, 23∶0, 24∶0 and 24∶1 SM and Cer species.

Finally we tested if the combination of certain lipid species increased their prognostic value. Therefore a quotient of the six most protective species (SM 23∶0, SM 24∶0, PC 38∶5, PC 38∶6, PC 36∶5 and PC 40∶6) and the six species with the strongest positive mortality association (SM 16∶0, SM 24∶1, Cer 16∶0, Cer 24∶1, PC 32∶0 and PC O-32∶0) was calculated. The result of that were hazard ratios of 2.725 (95% confidence interval: 2.350–3.160, p<0.001) for cardivascular and 2.508 (95% confidence interval: 2.223–2.830, p<0.001) for total mortality, which is far higher than the results for individual species. To compare the quality of the quotient with known risk factors, like age, gender, LDL, HDL, BMI, diabetes, hypertension, CAD and C-reactive protein (CRP), the individual hazard ratios (unadjusted) within our cohort were calculated (Table 2). Diabetes, CAD and age (per 10 years increase) showed an approximately 2-fold increase; hypertension, CRP and male sex showed an approximately 1.3-fold increase while female sex showed a roughly 1.3-fold decrease of risk (Table 2). LDL, HDL and BMI showed no effect (Table 2). The quotient of the sums of protective and risk lipid species on the other hand showed an almost 3-fold increased risk.

**Table 2:** Comparison of hazards ratios of the quotient of the sums of the protective and risk lipid species with known cardiovascular risk factors.

Risk factor	p-value	HR	95% CI
Lipid ratio (per 0.1 unit increase)	<0.001	2.886	2.580 – 3.227
Age (per 1 year increase)	<0.001	1.070	1.061 – 1.078
Age (per 10 year increase)	<0.001	1.972	1.821 – 2.135
Female gender	0.004	0.789	0.672 – 0.927
Male gender	0.004	1.267	1.078 – 1.488
LDL cholesterol	0.042	0.998	0.995 – 0.999
HDL cholesterol	<0.001	0.982	0.974 – 0.988
BMI	0.009	0.976	0.958 – 0.994
Diabetes	<0.001	2.273	1.973 – 2.618
Hypertension	<0.001	1.387	1.168 – 1.646
CAD (> = 20% stenosis)	<0.001	2.220	1.787 – 2.757
CAD (> = 50% stenosis)	<0.001	2.031	1.697 – 2.429
lnCRP	<0.001	1.304	1.237 – 1.372

BMI, body mass index; CAD, coronary artery disease; CI, confidence interval; CRP, C-reactive protein; HDL, high density lipoprotein; HR, hazard ratio; LDL, low density lipoprotein.

## Discussion

**Figure 4:**
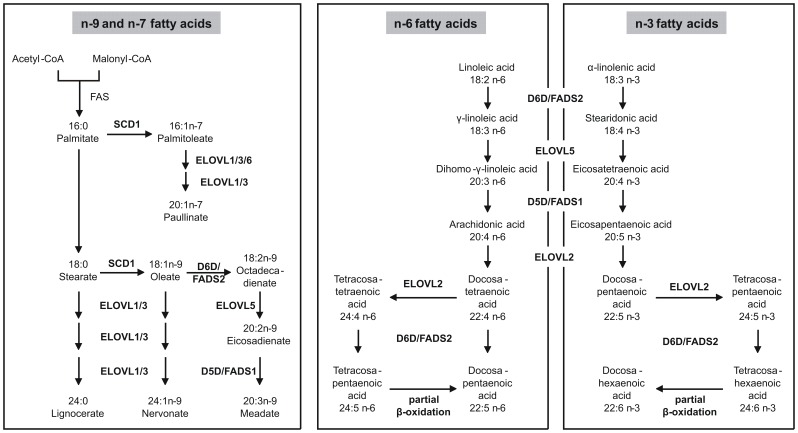
Elongation and desaturation of fatty acids. The n-9-series is endogenously derived from stearate. The n-7-series is endogenously derived from palmitate. Starting point for the n-6- and the n-3-series are linoleic acid and α-linolenic acid, respectively, which are not synthesized endogenously and must be taken up as essential fatty acids through the diet. Adapted from (14). D5D: delta-5 desaturase; D6D: delta-6 desaturase; ELOVL: elongation of long chain fatty acids; FAS: fatty acid synthase; SCD: stearoyl-CoA desaturase.

In this study we provide data that plasma glycerophospholipid and sphingolipid species are easily accessible predictors of mortality. Identified saturated and monounsaturated PC species are positively associated with mortality, with PC 32∶0 revealing the strongest positive association (Figure 1A). PC 32∶0 most likely is composed of two palmitates (16∶0) at sn-1 and sn-2 position, alternatively myristate (14∶0) and stearate (18∶0) could also form PC 32∶0. The delta-9-desaturase (D9D) stearoyl-CoA desaturase (SCD/FADS5/D9D) shows specific affinity for the two main dietary saturated FAs palmitate and stearate, converting them to the corresponding monounsaturated FAs palmitoleate (16:1n-7) and oleate (18:1n-9) (figure 4). This indicates a protective role of SCD as PC 32∶1 and PC 32∶2, containing SCD desaturation products, compared to PC 32∶0 reduce their mortality association, with PC 32∶2 being even slightly but significantly protective (Figure 1A). This is in agreement with studies suggesting that deregulation of SCD supports inflammation, atherosclerosis, hypertriglyceridemia, and metabolic syndrome [38]. Besides SCD/FADS5/D9D, the delta-6 desaturase (D6D)/FADS2 may also be involved, generating octadecadienate (18:2n-9) from oleate. This effect is less pronounced for PC 36∶2 and PC 38∶2, although a similar tendency is visible.

10 PC species containing long-chain PUFAs were found associated with a protective effect in our analysis (Figure 1A). This may reflect diet and/or physical activity of the patients, as it was shown that e.g. fish oil supplementation increased the relative amount of PUFA containing phospholipids and tryglycerides [39] and that physical activity leads to higher amounts of PUFA containing TGs [40]. These PC species probably contain FAs originating from n-3 and/or n-6 dietary essential FAs, and their elongation/desaturation products. Elongation/desaturation products of n-9 octadecadienate could also be components of these PC species. It may be speculated that the protective effect is based on either (i) withdrawing pro-inflammatory eicosanoids of the n-6 series from their signaling pathways or (ii) providing anti-inflammatory eicosanoids of the n-3 series. In summary, PC species containing long chain saturated and monounsaturated n-9 FAs positively associate with mortality while long-chain PUFAs appeared to be associated with a protective effect. Although it is not possible to resolve the exact FA composition of the measured PC species with the method used here, it is highly probable that the protective species contain arachidonic acid (20:4n-6). This is supported by a previous study observing that an increased 20:4n-6 to 20:3n-6 ratio attends with a reduced coronary heart disease risk [41].

Elongation steps of the n-3 and n-6 series are catalyzed by members of the elongation of very long chain fatty acids-like (ELOVL) family, namely ELOVL2 and ELOVL5 (figure 4). ELOVL2 catalyzes the rate-limiting PUFA elongation steps of n-6 arachidonyl-CoA (20∶4) to docosatetraenoyl-CoA (22∶4), and further to tetracosatetraenoyl-CoA (24∶4) (figure 4). ELOVL2 is also involved in the n-3 pathway to form docosapentaenoyl-CoA (22∶5) and tetracosapentaenoyl-CoA (24∶5) (figure 4). Strongest association of ELOVL2 was shown with ratios between PCs that differ by two or four carbon atoms in their FA side chains, and that are likely to contain a PUFA of the aforementioned type at the sn-2 position of their glycerol backbone [15]. Desaturation is mediated by D5D/FADS1 and D6D/FADS2. D5D/FADS1 is required for the desaturation of 20:3n-6, 20:4n-3 and 20:2n-9 FAs, leading to 20:4n-6, 20:5n-3 and 20:3n-9 FAs (figure 4). All substrates and products of the D5D/FADS1 reaction are very likely constituents of PC 38∶5. D6D/FADS2 uses 18:2n-6 and 18:1n-9 as substrates (figure 4), which also represent potential constituents of PC 38∶5. Desaturation of 18:1n-9 generates 18:2n-9, which also could be a constituent of PC 38∶5 (figure 4). The protection-associated PC species 36∶4, 38∶4, 36∶5 and 38∶6 were previously shown to negatively correlate with an intronic SNP (rs174548) in the D5D/FADS1 gene, while PC 34∶2, which we found mortality associated, revealed a positive correlation with this SNP ([16] and references therein).

LPC species originate predominantly from phospholipase A_1_/A_2_, endothelial lipase and lecithin-cholesterol acyltransferase (LCAT) activity [9]. Cleavage can occur at either the sn-1 (phospholipase A_1_) or sn-2 (phospholipase A_2_) position of the glycerophospholipid. Three LPC species with mortality association were identified (LPC 16∶0, LPC 18∶0 and LPC 18∶2) which are all associated with a protective effect (Figure 1B). 16∶0 and 18∶0 FAs are equally found in PC species positively and negatively associated with mortality. Recently it was shown that circulating LPC species are reduced in obesity and that more probably diet and adiposity than IR and diabetes are the reason for this [42]. Similar to the protection-associated PC species, the protection-associated LPC species may reflect diet.

In contrast to PC species all identified PC O (Figure 1C), PE and PE O (Figure 2A and B) species besides PE 36∶6 and PE O 38∶7 showed positive mortality association independent from chain length and degree of saturation. Interestingly similar tendencies were observed for 16∶0, 23∶0, 24∶0 and 24∶1 SM and Cer (Figure 3A and B), stressing the importance of SM/Cer conversion. SM is converted to Cer via the sphingomyelin phosphodiesterases SMPD1-4 [43] and ectonucleotide pyrophosphatase/phosphodiesterase 7 (ENPP7) [44]. The sphingomyelin synthases SGMS1-2 are capable of catalyzing both, the conversion SM to Cer and Cer to SM {Huitema, 2004 4513/id}. The mortality associated Cer species 16∶0 and 24∶1 together with the protection-associated Cer 23∶0 and Cer 24∶0 were shown to correlate with a SNP (rs680379) upstream the SPTLC3 gene [14]. While Cer 16∶0 revealed negative correlation with SPTLC3 the longer Cer species showed a positive correlation [14]. From the 9 SM species identified in this work 5 were shown to correlate with SNPs in the SGPP1 region [14].

The observed hazard ratios of 2.725 (95% confidence interval: 2.350–3.160, P<0.001) for cardivascular and 2.508 (95% confidence interval: 2.223–2.830, P<0.001) for total mortality were achieved using a quotient of the sums of the six most protective species (SM 23∶0, SM 24∶0, PC 38∶5, PC 38∶6, PC 36∶5 and PC 40∶6) and the sums of the six species with the strongest positive mortality association (SM 16:0, SM 24∶1, Cer 16∶0, Cer 24∶1, PC 32∶0 and PC O-32∶0). They are independent of the known cardiovascular risk factors age, gender, smoking, LDL, HDL, diabetes and hypertension. In comparison the quotient of protective and risk lipid species had an even better prognostic value than the known risk factors in our cohort (Table 2). The surprising result that LDL did not affect the risk of cardiovascular mortality is probably an effect of LDL lowering therapy.

In summary the data presented here indicate together with previously published SNP lipid species-correlations that SNPs in lipid metabolism related gene-region influence lipid species levels and mortality. Moreover, the quotient of the sums of the six most protective species and the six species with the strongest positive mortality association may be a valuable prognostic marker, which has to be confirmed in independent cohorts.
